# A Nanostructured Matrices Assessment to Study Drug Distribution in Solid Tumor Tissues by Mass Spectrometry Imaging

**DOI:** 10.3390/nano7030071

**Published:** 2017-03-21

**Authors:** Silvia Giordano, Valentina Pifferi, Lavinia Morosi, Melinda Morelli, Luigi Falciola, Giuseppe Cappelletti, Sonja Visentin, Simonetta A. Licandro, Roberta Frapolli, Massimo Zucchetti, Roberta Pastorelli, Laura Brunelli, Maurizio D’Incalci, Enrico Davoli

**Affiliations:** 1 Environmental Health Sciences Department, Mass Spectrometry Laboratory, IRCCS Istituto di Ricerche Farmacologiche Mario Negri, Via La Masa 19, 20156 Milano, Italy; silvia.giordano@marionegri.it (S.G.); roberta.pastorelli@marionegri.it (R.P.); laura.brunelli@marionegri.it (L.B.); 2 Dipartimento di Chimica, Università degli Studi di Milano, Via Golgi 19, 20133 Milano, Italy; valentina.pifferi@unimi.it (V.P.); melinda.morelli@unimi.it (M.M.); luigi.falciola@unimi.it (L.F.); giuseppe.cappelletti@unimi.it (G.C.); 3 Oncology Department, Cancer Pharmacology Laboratory, IRCCS Istituto di Ricerche Farmacologiche Mario Negri, Via La Masa 19, 20156 Milano, Italy; lavinia.morosi@marionegri.it (L.M.); simonettaandrea.licandro@marionegri.it (S.A.L.); roberta.frapolli@marionegri.it (R.F.); massimo.zucchetti@marionegri.it (M.Z.); maurizio.dincalci@marionegri.it (M.D.); 4 Department of Molecular Biotechnology and Health Science, University of Torino, Via Nizza 52, 10126 Torino, Italy; sonja.visentin@unito.it

**Keywords:** mass spectrometry imaging, MALDI, TiO_2_, gold nanoparticles, halloysite, cancer imaging

## Abstract

The imaging of drugs inside tissues is pivotal in oncology to assess whether a drug reaches all cells in an adequate enough concentration to eradicate the tumor. Matrix-Assisted Laser Desorption Ionization Mass Spectrometry Imaging (MALDI-MSI) is one of the most promising imaging techniques that enables the simultaneous visualization of multiple compounds inside tissues. The choice of a suitable matrix constitutes a critical aspect during the development of a MALDI-MSI protocol since the matrix ionization efficiency changes depending on the analyte structure and its physico-chemical properties. The objective of this study is the improvement of the MALDI-MSI technique in the field of pharmacology; developing specifically designed nanostructured surfaces that allow the imaging of different drugs with high sensitivity and reproducibility. Among several nanomaterials, we tested the behavior of gold and titanium nanoparticles, and halloysites and carbon nanotubes as possible matrices. All nanomaterials were firstly screened by co-spotting them with drugs on a MALDI plate, evaluating the drug signal intensity and the signal-to-noise ratio. The best performing matrices were tested on control tumor slices, and were spotted with drugs to check the ion suppression effect of the biological matrix. Finally; the best nanomaterials were employed in a preliminary drug distribution study inside tumors from treated mice.

## 1. Introduction

In drug discovery and development it is important to understand the pharmacokinetics, investigating the absorption, distribution, metabolism, and excretion (ADME) of molecules. Several analytical methods, based on high-performance liquid chromatography (HPLC) and Liquid chromatography tandem-mass spectrometry (LC-MS/MS), have been developed and employed on plasma and tissue homogenates to establish drug concentration profiles of drugs [1]. Classical pharmacokinetic studies measure the average drug accumulation in tissues assuming a homogeneous distribution of the molecule and ignore organ compartmentalization. However, the manner in which a compound and its metabolites localize and distribute inside a tissue is important in order to ascertain whether they reach the intended target site. This is particularly true in oncology where the data obtained may reveal very little about the drug’s ability to penetrate the tumor, because they neglect the information about the structure of the tumor microenvironment resulting in a loss of information about spatial distribution [2,3]. In particular, the imaging of drugs inside biological tissues is pivotal in oncology to assess whether a drug reaches high enough concentrations in all tumor cells and to develop new strategies to improve penetration and the outcome of chemotherapy [4].

In recent decades, several imaging techniques have been developed to investigate the distribution of compounds inside a tissue, such as magnetic resonance imaging (MRI), positron emission tomography (PET), or wall body autoradiography (WBA), and are routinely used in clinical diagnosis [5,6,7,8,9,10].

Mass spectrometry imaging (MSI) is one of the latest, rapidly growing surface analysis techniques for the detection, localization, and identification of molecules in tissues [11,12,13]. MSI permits the simultaneous visualization of multiple endogenous or exogenous compounds with high specificity and sensitivity, without the need for radiolabeled compounds [14,15]. It was developed in the late 1970s with the development of laser microprobe mass spectrometry (LMMS) and laser microprobe mass analysis (LAMMA) [16]. These techniques use a focused laser beam that generates a light pulse to desorb and ionize molecules inside solid samples. Ionized molecules are excited and accelerated into a time of flight (TOF) mass analyzer, particularly suitable for the imaging of small molecules (M_W_ < 10,000 Da). Laser desorption/ionization based techniques have always been the most widely used in mass spectrometry for imaging [17] and they were soon implemented with the introduction of organic matrices to improve the detection of higher mass species, leading to the development of Matrix Assisted Laser Desorption Ionization (MALDI).

MALDI is the prevailing MSI technique used in pre-clinical and clinical research [11,18] to obtain information about biomolecules over a wide mass range, in living cells and tissues [19]. Originally developed for the imaging of molecules with high molecular weights like proteins and peptides [20,21], it is now also used to visualize the distribution of small molecules (M_W_ < 1000 Da) like drugs and metabolites as well [14,22,23,24,25].

The principle behind MALDI-MSI is to acquire a mass spectrum for each point of a tissue section by rastering a laser beam at defined geometrical coordinates. The UV laser energy is absorbed by a matrix present in a homogeneous layer on the tissue slice that facilitates analyte extraction in the MALDI ion source [26,27]. The choice of the suitable matrix for the molecule of interest is primarily driven by empirical results and is critical during the development of a MALDI-MSI protocol, since matrix ionization efficiency can change depending on the analyte structure and its physicochemical properties. Typically used matrices for MALDI experiments (organic compounds such as sinapinic acid, 2,5-dihydroxybenzoic acid, or a-cyano-4-hydroxycinnamic acid) are inadequate for small molecule imaging because of their excessive fragmentation and high background noise in the low mass region that can mask the analyte ion signal, and also because of their co-crystallization with the analyte that can affect the spatial resolution [14]. For these reasons the study of MALDI matrices is continuously in progress and recently, besides the advances in nanotechnologies, several new compounds have been tested as possible matrices to enhance desorption/ionization [28].

A different approach based on the use of inorganic fine particles takes advantage of their physicochemical properties such as high photo-adsorption, low heat capacity, and large surface area. This ensures rapid heating and highly localized and uniform energy deposition, resulting in efficient sample desorption and ionization [29], allowing for the detection of large intact molecules. The great advantage of the use of nanoparticles as MALDI matrices is the almost complete absence of background signals from matrix degradation that made them suitable for small molecule imaging studies. Another positive aspect is that the spatial resolution only depends on the instrumental specifications such as laser spot diameter and not on the matrix crystal dimensions [14]. Among several nanomaterials, we tested gold and titanium nanoparticles, and halloysites and carbon nanotubes as possible inorganic MALDI matrices.

The use of gold and platinum nanostructures in laser desorption/ionization (LDI) MS of low molecular weight compounds was reviewed by Bergman et al. [30]. Gold nanoparticles (AuNPs) have received attention for their suitability in surface assisted laser desorption ionization mass spectrometry (SALDI-MS), where these inorganic substrates offer large surface areas, simple sample preparation techniques, flexibility, and selectivity of sample deposition [31].

Titanium dioxide can be considered as a matrix for MALDI, since the laser wavelength falls in the range of its absorption band. In particular, the literature reports some examples of the use of this material for the determination of carbohydrates [32] and antitumor drugs [22,33].

Halloysite (HNT), a naturally occurring aluminosilicate nanotube (Al_2_Si_2_O_5_(OH)_4_·2H_2_O), is a two-layered aluminosilicate, with a predominantly hollow tubular structure. Chemically, the outer surface of the HNTs has properties similar to SiO_2_, while the inner cylinder core is related to Al_2_O_3_. In a recent paper, halloysite nanoclay showed good potential as a SALDI surface for the rapid analysis of low molecular mass polyesters and their degradation products [34].

Carbon nanotubes (CNTs) are allotropes of carbon with a cylindrical nanostructure. They have been reported to be an effective MALDI matrix for small molecules [35], eliminating the interfering matrix peaks and forming a web morphology to fully disperse the analyte and allow for strong ultraviolet absorption for enhanced pulsed laser desorption and ionization.

We compared the ability of these five nanostructured matrices to ionize six different anticancer drugs: taxans (paclitaxel, PTX and ortataxel, OTX), tyrosine kinase inhibitors (imatinib, IMT and lucitanib, LCT), an antineoplastic antibiotic (doxorubicin, DOXO), and a DNA binding protein (trabectedin, ET) [36,37]. All nanomaterials were firstly screened to compare their ionization efficiency by co-spotting them with equimolar concentrations of the drugs directly on the MALDI plate. We therefore evaluated the drug signal intensity, expressed as the signal to-noise ratio (S/N), considering both the matrix degradation effects that lead to excessive background chemical noise and the instrumental contamination that prevents the effective use of nanomaterials. The best performing matrices were tested on control tumor slices spotted with drugs, to assess the influence of the biological matrix on the intensity of the analytes’ ion signal. Finally, the best nanomaterials were applied in a preliminary drug distribution study inside tumors harvested from treated mice.

The main objective of this study is the improvement of the MALDI-MSI technique in the field of pharmacology, developing specifically designed surfaces for the imaging of different anticancer drugs, with high spatial resolution, sensitivity, and reproducibility.

## 2. Results

**Figure 1:**
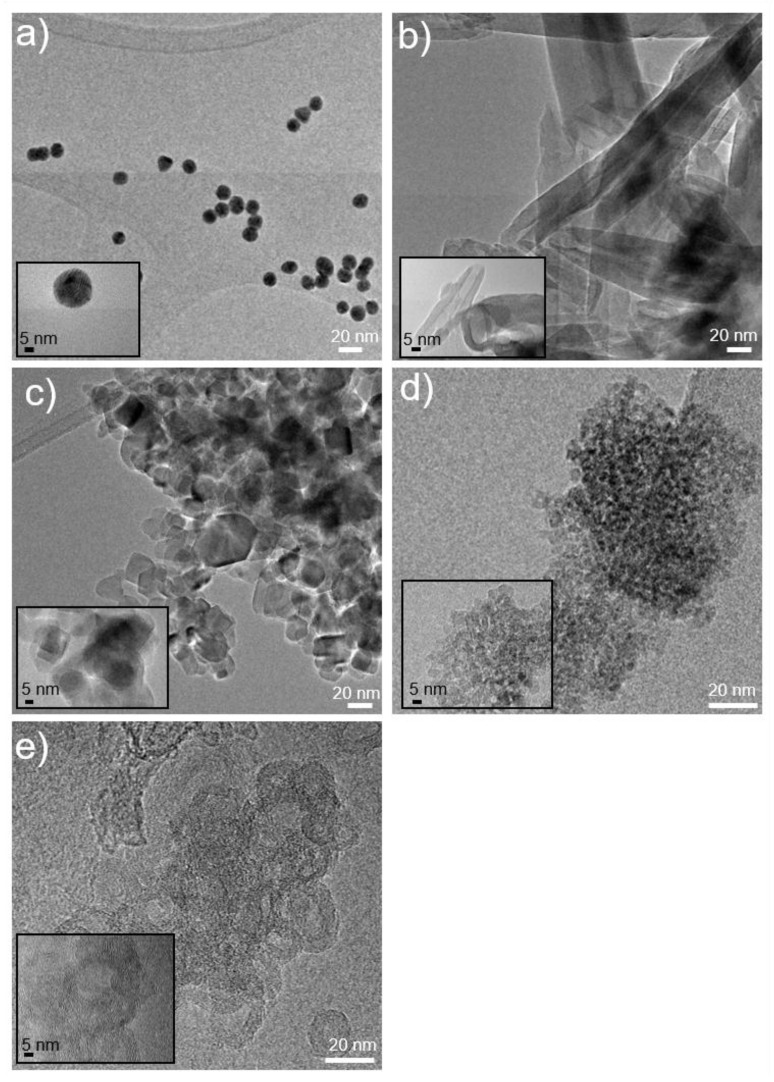
Transmission electron microscope (TEM) images of (**a**) Au nanoparticles (AuNPs); (**b**) Halloysite nanotubes (HNTs); (**c**) TiO_2_ P25 nanoparticles; (**d**) TiO_2_ Hombikat nanoparticles UV100 and (**e**) Carbon nanotubes (CNTs) at different magnifications (scale bars correspond to 20 nm and 5 nm).

All nanostructured materials were deeply characterized by the morphological, structural, and surface points of view, by ultraviolet-visible spectroscopy (UV), transmission electron microscope (TEM), scanning electron microscope (SEM), and dynamic light scattering (DLS) analyses. Relevant features are reported in Table 1. Figure 1 shows TEM images of the nanoparticles.

**Figure A1:**
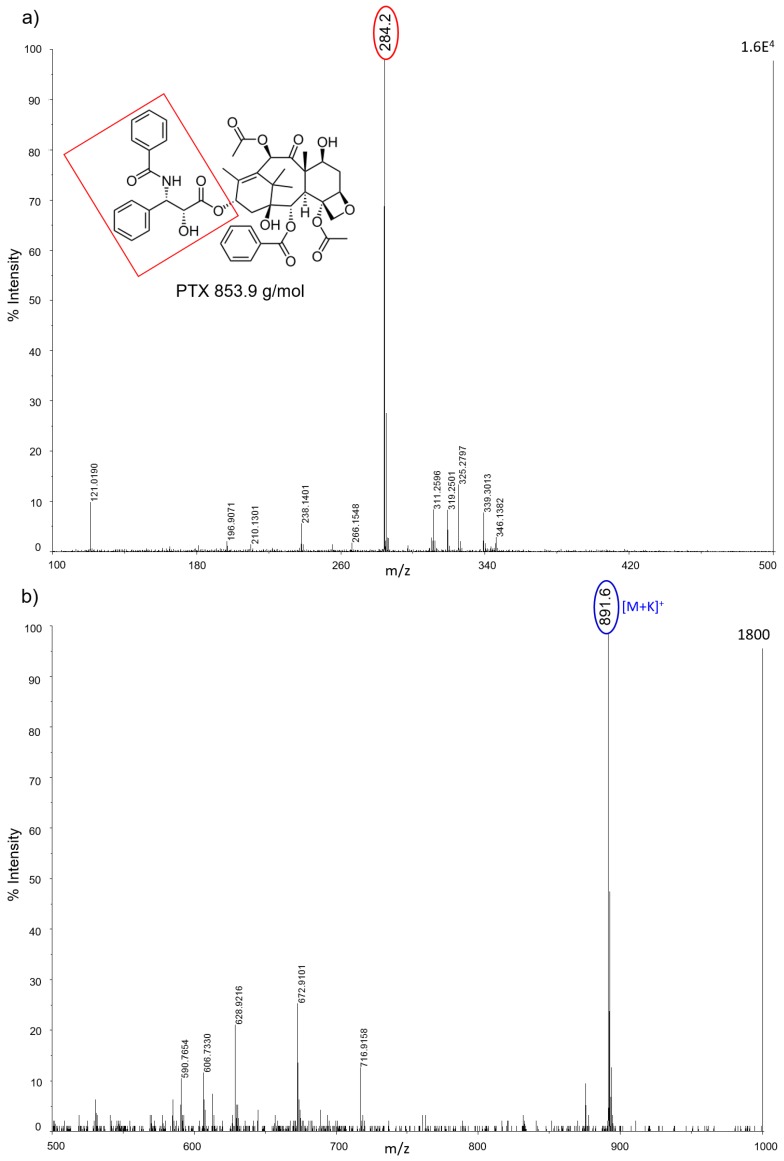
PTX mass spectra examples in (**a**) reflectron negative ion mode with the relative molecular structure and fragment detected using TiO_2_ P25 nanoparticles and (**b**) reflectron positive ion mode using TiO_2_ Hombikat nanoparticles.

**Figure A2:**
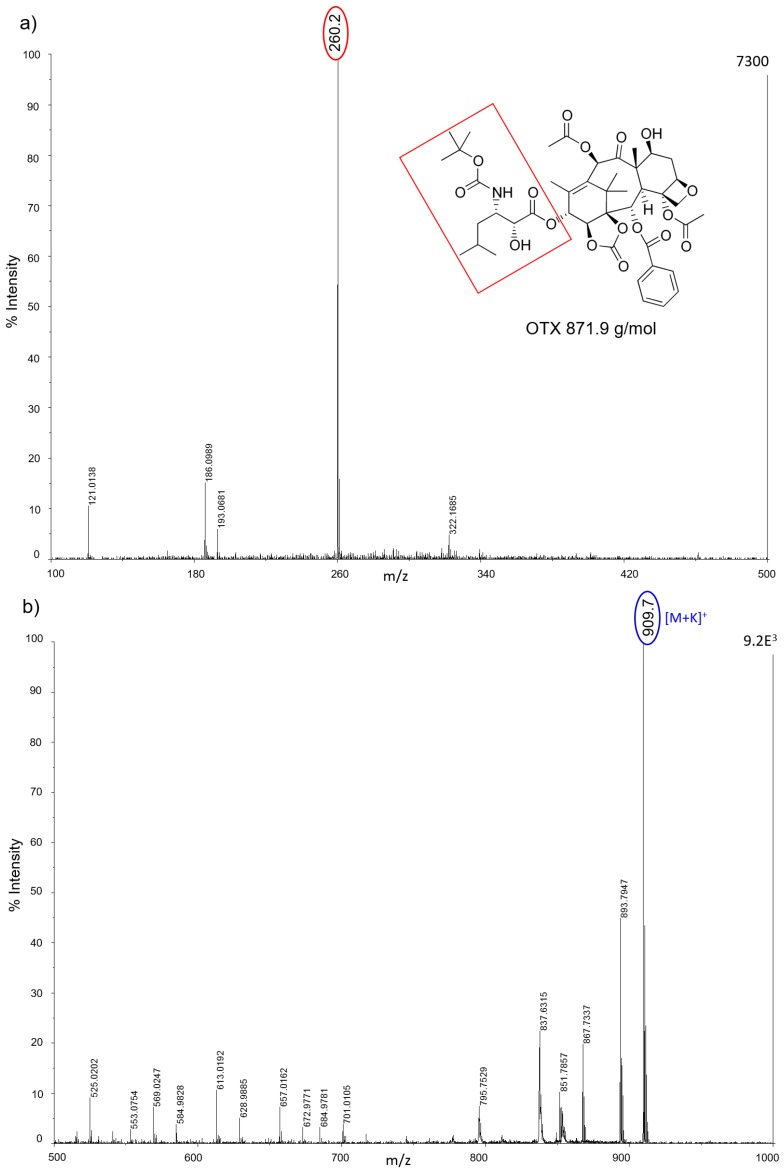
OTX mass spectra examples in (**a**) reflectron negative ion mode with the relative molecular structure and fragment detected using TiO_2_ P25 nanoparticles and (**b**) reflectron positive ion mode using TiO_2_ P25 nanoparticles.

**Figure A3:**
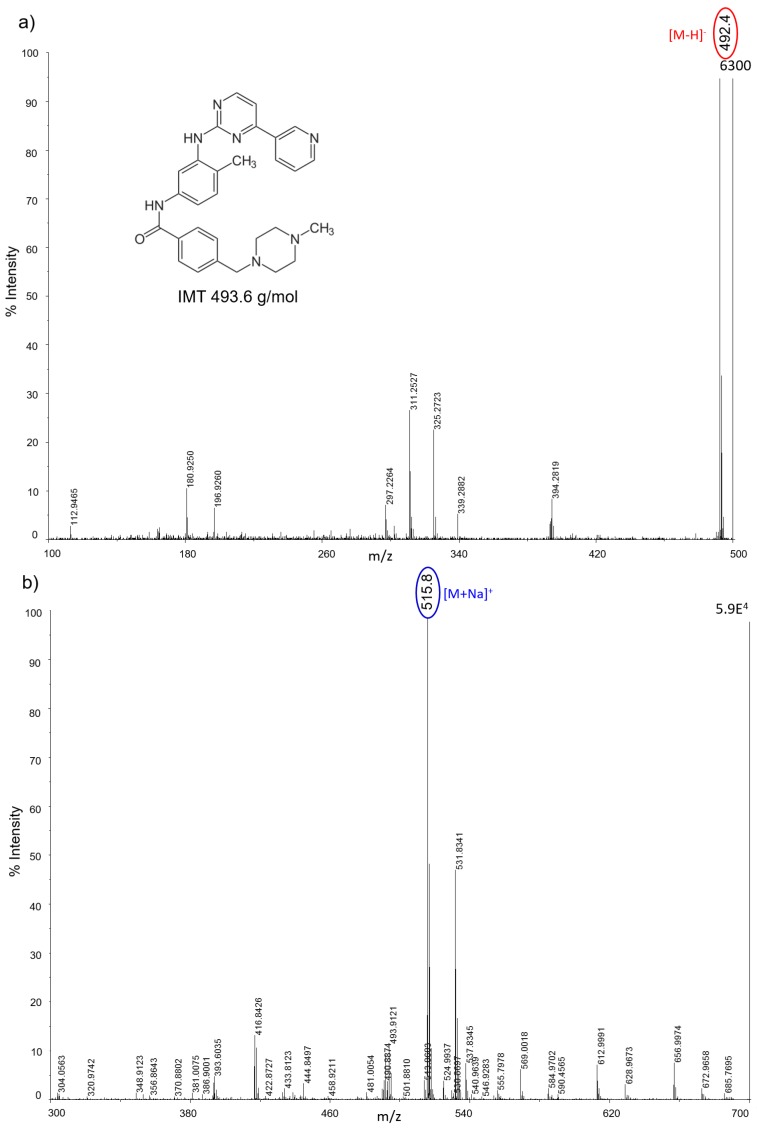
IMT mass spectra examples in (**a**) reflectron negative ion mode with the relative molecular structure and fragment detected using Halloysite matrix and (**b**) reflectron positive ion mode using gold nanoparticles.

**Figure A4:**
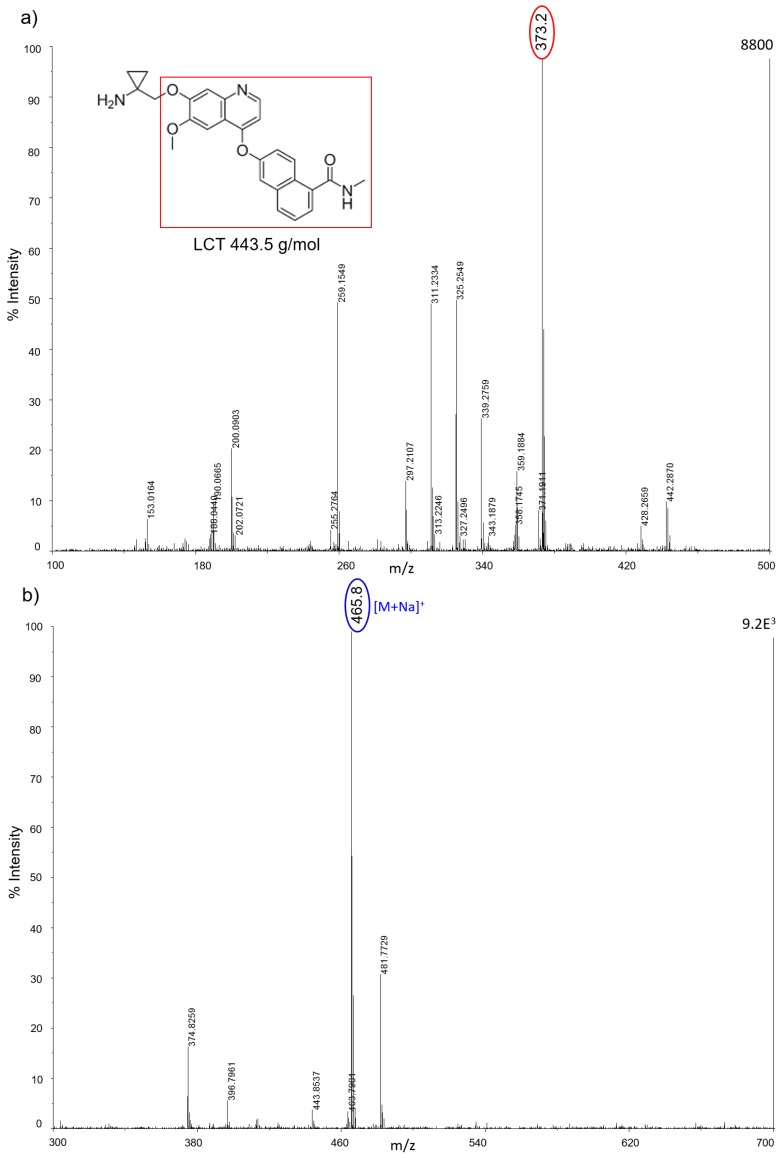
LCT mass spectra examples in (**a**) reflectron negative ion mode with the relative molecular structure and fragment detected using halloysite matrix and (**b**) reflectron positive ion mode using halloysite matrix.

**Figure A5:**
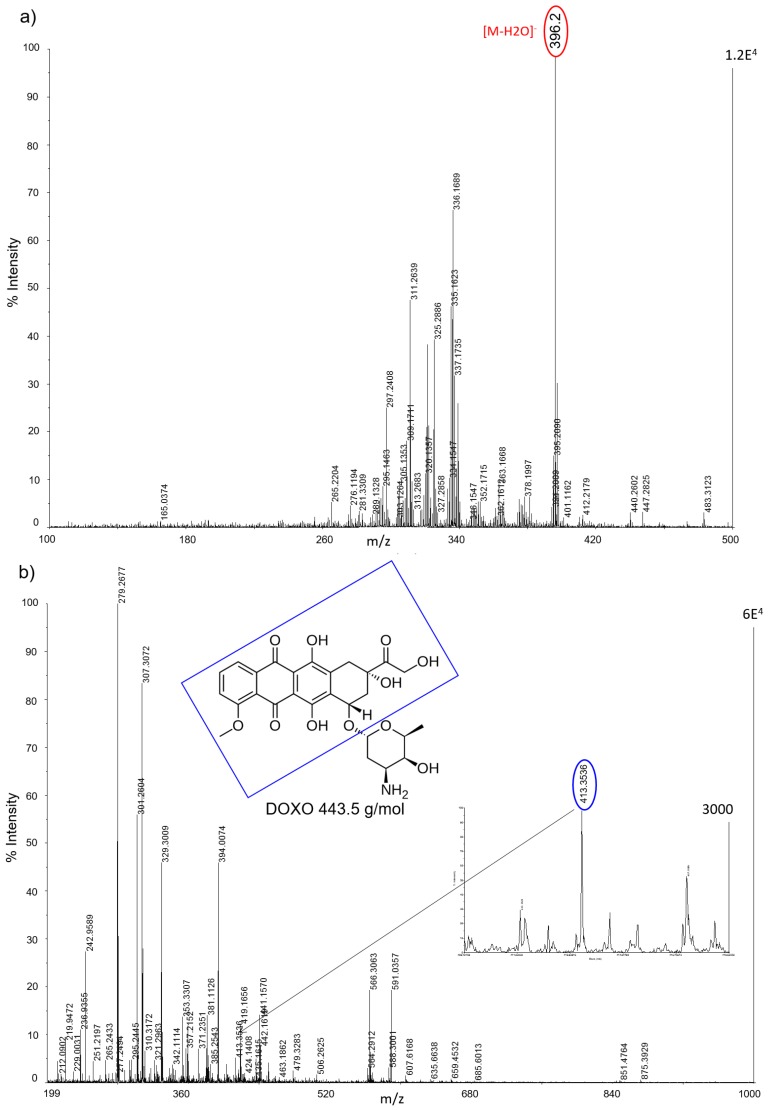
DOXO mass spectra examples in (**a**) reflectron negative ion mode with the relative molecular structure and fragment detected using TiO_2_ Hombikat nanoparticles and (**b**) reflectron positive ion mode using TiO_2_ Hombikat nanoparticles.

**Figure A6:**
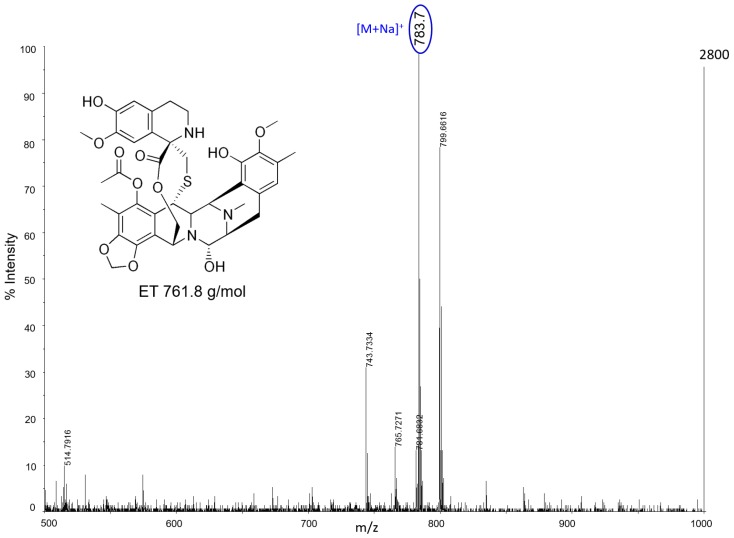
ET mass spectra example in reflectron positive ion mode using TiO_2_ Hombikat nanoparticles.

All nanostructured matrices were firstly screened by co-spotting them with equimolar concentrations of six anticancer drugs on the MALDI plate and evaluating both the signal intensity and the S/N for each combination of drug and matrix. The detected MS fragments for each drug are reported in Figure A1, Figure A2, Figure A3, Figure A4, Figure A5 and Figure A6.

**Figure 2:**
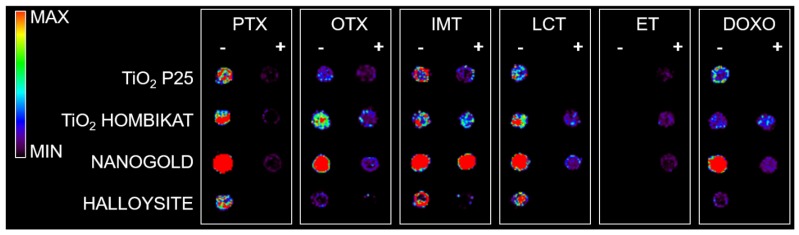
Drug standards (100 pmol/µL) spotted on the matrix-sssisted laser desorption/ionization MALDI plate with different nanostructured materials. For each drug, the acquisition in negative ion mode is shown on the left and the acquisition in positive ion mode is shown on the right of each column. The negative ion mode gives more intense signals for almost all drugs except for trabectedin (ET), which was detectable only in positive ion mode.

Almost all drugs were successfully ionized both in negative and positive ion modes using gold, halloysite, and titanium, while trabectedin (ET) was detectable only in positive ion mode at *m*/*z* 743 after losing a water molecule (Figure 2). P25 and Hombikat TiO_2_ nanoparticles were particularly suitable to visualize the two taxans, PTX (*m*/*z* 284) and OTX (*m*/*z* 271) in negative ion mode, but they also allowed the ionization of smaller molecules like IMT (*m*/*z* 492) and LCT (*m*/*z* 373). AuNPs gave more intense signals, especially in positive ion mode for the tyrosine-kinase inhibitor IMT as a sodium adduct at *m*/*z* 515. Compared to TiO_2_ nanoparticles, AuNPs were more efficient for ionizing and visualizing smaller molecules such as IMT and LCT, giving a more intense signal in general. HNTs gave good visualization only for three of the six drugs: PTX, IMT, and LCT. CNTs efficiently ionized all drugs both in negative and positive ion modes, but could not be used for imaging.

**Figure 3:**
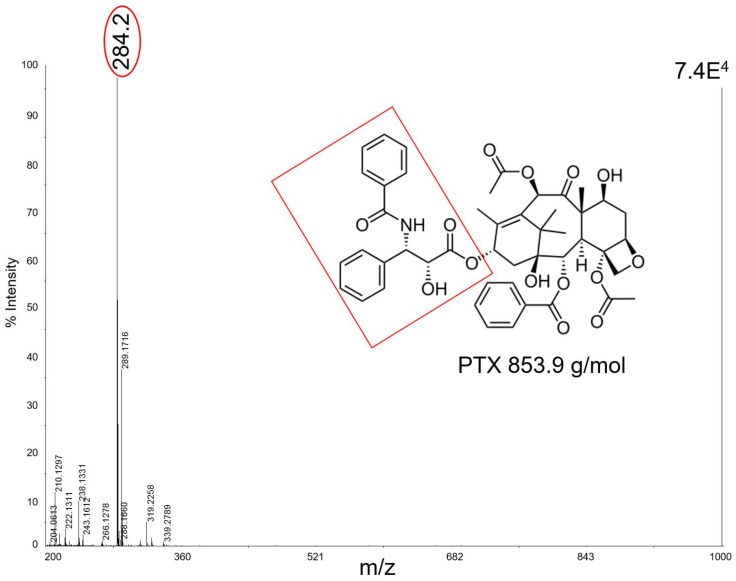
MALDI mass spectrum of paclitaxel (PTX, 50 pmol) in negative ion mode using CNTs. The fragment ion *m*/*z* 284 is clearly observable in the spectrum with a high S/N ratio.

Figure 3 shows, as an example, the mass spectrum of 50 pmol of PTX which in negative ion mode with CNTs fragments to produce ions at *m*/*z* 284 as a base peak. In this mass region the spectrum has a very high S/N, suggesting a good effectiveness of the technique with this kind of matrix. Unfortunately, CNTs tended to fly off from the target plate when subjected to the laser pulse, contaminating the ion source and interfering with the instrument functionality.

**Figure 4:**
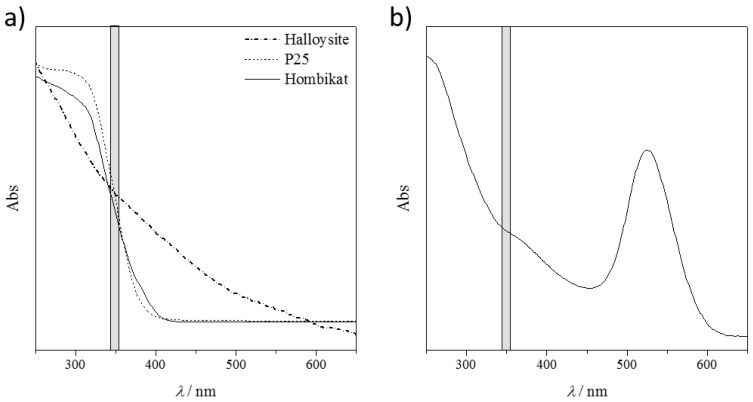
UV-Vis absorption behavior of the adopted nanostructured materials. The grey shadow represents the laser emission wavelengths. (**a**) Diffuse reflectance spectra (DRS) of halloysite and titanium nanomaterials; (**b**) Absorption spectra of AuNP slurry.

The different performances of the nanomaterials towards the ionization of the drug molecules may be ascribed to many factors, starting with the physico-chemical properties (Table 1), their ability of energy sorption from the laser source, and transfer to the analytes. Figure 4a shows the diffuse reflectance spectra (DRS) of the tested solid nanomaterials and Figure 4b shows the absorption spectra of the AuNP slurry. Non-conducting HNTs presented no absorption peak in the explored UV-Vis range, particularly in the laser emission wavelengths (grey shadow in Figure 4). This resulted in a poor performance of these materials in visualizing the drug fragments.

Both titanium samples, instead, showed a strong absorption (inflection point of the curves) particularly in the laser emission range. However, not all the absorbed energy could be transferred to the target molecules, as these materials are semiconductors. This yields a good compromise between the absorbed and released energy, with promising results in ionizing almost all the bigger drug molecules. Unfortunately, the released energy is still so high that the small fragments are not detected because of their complete mineralization (no signals are detectable in the low MS region, *m*/*z* < 100).

The best performing material seems to be the AuNPs. The UV-Vis relative absorption lies far from the laser absorption and there is only a small shoulder in the desired range (Figure 4b). The partial energy absorption is enough to ionize both the large and the small molecules (as it is a conductive material), but not sufficient to completely destroy them. However, by considering the same nanomaterial matrix, different ionization results are obtained by varying the chemical nature of the analyte, and thus their interaction/adsorption onto the solid support. Further studies are required to show which material is the best one for the target molecule.

**Figure 5:**
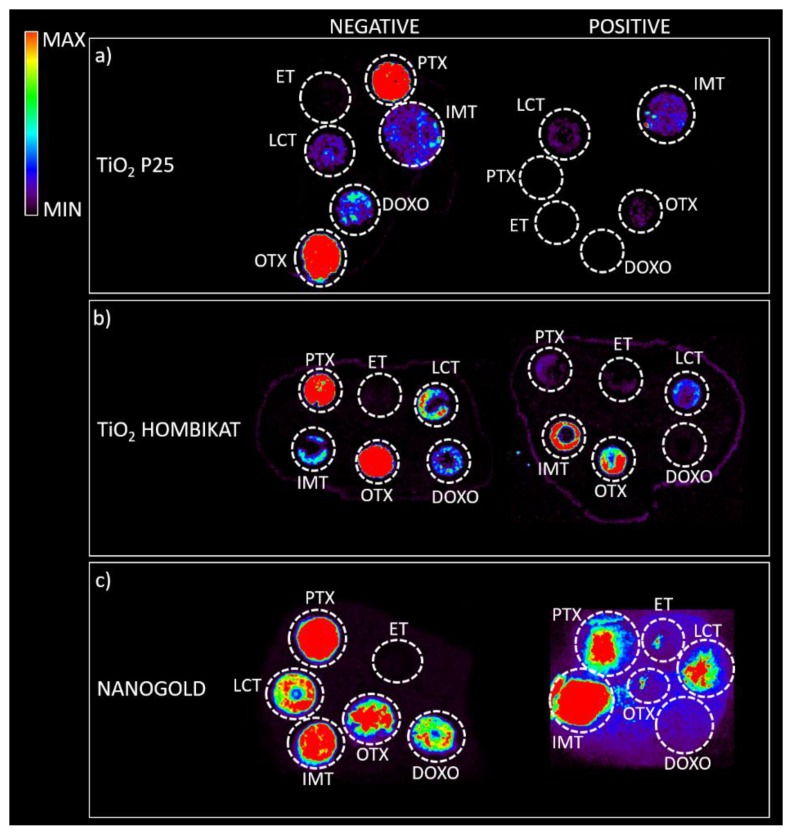
Control tumor tissues treated with a vehicle, spotted with 100 pmol/µL drug standards, then sprayed with different matrices. Acquisitions were performed with a spatial resolution of 100 µm both in negative ion mode (left) and positive ion mode (right). (**a**) TiO_2_ P25 nanoparticles allow desorption and ionization mainly for PTX (*m*/*z* 284) and OTX (*m*/*z* 260) in negative ion mode; (**b**) TiO_2_ Hombikat efficiently ionizes the two taxans PTX and OTX in negative ion mode but also imatinib (IMT) and OTX in positive ion mode; (**c**) Gold nanoparticles allow the visualization of almost all the drugs spotted on the control tissue both in negative and positive ion mode. Moreover, with gold nanoparticles it is possible to also visualize DOXO in negative ion mode with a good signal.

Based on the present findings, the most promising nanomaterials (P25 TiO_2_, Hombikat TiO_2_, and particularly AuNPs) were tested to assess how the biological matrix influenced the intensity of the analyte ion signal and for interfering signals by spotting drug standards on control tumor tissue slices that were then sprayed with each matrix for imaging experiments (Figure 5). The results on the tissues confirm the previous screening on the plates: TiO_2_ P25 nanoparticles allowed desorption and ionization mainly for PTX (*m*/*z* 284) and OTX (*m*/*z* 260) in negative ion mode; TiO_2_ Hombikat efficiently ionized the two taxans PTX and OTX in negative ion mode but also IMT and OTX in positive ion mode. AuNPs efficiently visualized almost all drugs (especially PTX and IMT) spotted on tissue (Figure 5c), but gave a low S/N ratio in positive ion mode. Finally, the HNTs matrix caused strong signal suppression when sprayed on tissues with the airbrush and was therefore excluded from further experiments.

**Figure 6:**
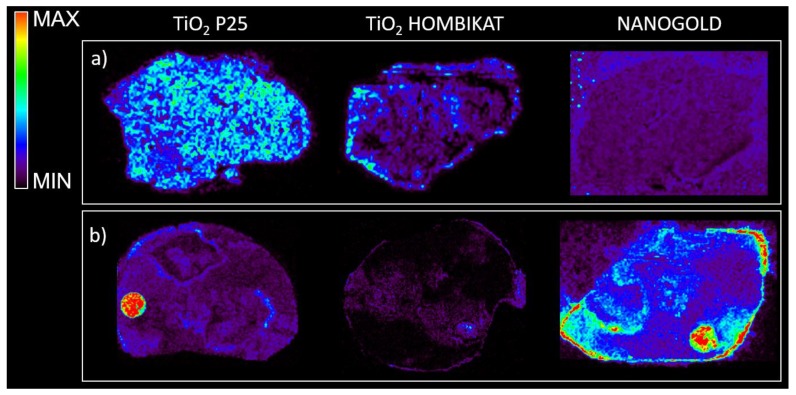
(**a**) MS ion images of PTX (*m*/*z* 284) in negative ion mode inside the same tumor tissue treated with 60 mg/kg intravenously (i.v.) of the drug with different matrices; (**b**) Imatinib (*m*/*z* 516) in positive ion mode inside the same tumor tissue treated at the dose of 400 mg/kg per os (p.o.) with different matrices.

Based on the results obtained from the control tissues spotted with the drug standards, TiO_2_ and AuNPs were selected to study the drug distribution inside tumors harvested from mice bearing xenografts treated with a single dose of drugs (60 mg/kg intravenously (i.v.) for PTX, 400 mg/kg per os (p.o.) for IMT, 20 mg/kg p.o. for LCT). Figure 6 shows that TiO_2_ based matrices appear to be suitable for visualizing the PTX distribution inside treated tissues in negative ion mode with high sensitivity, but did not give good visualization of IMT in negative or positive ion mode. In contrast, the nanogold-based matrix allowed a better visualization of the IMT distribution, highlighting its peripheral localization inside treated mesothelioma (even though there is high background noise). Both TiO_2_ and gold nanoparticles were not suitable for LCT distribution studies inside treated tumors.

## 3. Discussion

The imaging of drugs inside biological tissues is pivotal in oncology to understand how a compound and its metabolites are localized and distributed inside a tissue, in order to check that they reach the intended target site. Therefore, there is a growing need for methods to assess whether a drug reaches all tumor cells in adequate concentrations and to develop new strategies to improve penetration and the outcome of chemotherapy [38]. In this field, MSI is acquiring an important role thanks to its superior specificity and sensitivity [39]. However, a major shortcoming in setting up a MALDI-MSI method is the choice of a suitable matrix to guarantee efficient and reproducible ionization with low background noise. To address this problem, we screened different nanomaterials for the imaging of small molecules in biological tissues. Nanoparticles were suitable as MALDI matrices for small molecule imaging because of their uniform deposition over tissues (increasing lateral resolution) and the lack of background signals from matrix degradation. TiO_2_ nanoparticles have been previously reported to be suitable for MALDI mass spectrometry analysis of low molecular weight compounds, with almost complete absence of background noise [40], and recently gold based nanoparticles have been reported to assist laser desorption/ionization, avoiding co-crystallization of the analyte and the matrix [41,42]. For the synthesis of AuNPs, we used a classical chemical reduction method that has been extensively used in the preparation of nanoparticles because this method is simple, cheap, and can be used to prepare large quantities of nanoparticles with an accurate shape and dimensions. Among the conventional methods of synthesis of AuNPs by reduction of gold (III) derivatives, the most popular is that using citrate reduction of HAuCl_4_ in water, which was introduced by Turkevitch and revisited by Kimling [43,44].

We tested common nanoparticles with different features with six types of drugs, currently used as anticancer drugs in clinical settings. While the carbon nanotubes based matrix has been reported to provide higher detection sensitivity than classical organic matrices [35], and it enhanced the ionization of certain analytes of our interest, it was excluded from MSI applicability because of the marked instrumental contamination.

PTX efficiently ionizes with titanium based matrices which were however less suitable for the ionization of a smaller molecule such as IMT. AuNPs were convenient for the ionization and for the imaging of IMT, enlarging our ability to visualize different kind of molecules.

These results confirm that there is not a single type of matrix that suits all drugs. Further studies are needed to clarify the interactions between matrix and analyte to define case-by-case how to choose the best matrix and the best fitting combination that gives high sensitivity and therefore a good detection of drug distribution in the imaging of molecules inside biological tissues.

Further studies are also needed to accurately understand the mechanism of interaction between the matrix and analyte. Determining out why a particular matrix is more appropriate for the imaging of a certain drug could give clues for developing of new nanomaterials with designed texture for the imaging of a larger group of small molecules, to investigate drug distribution in primary tumors or metastases, while also offering new opportunities for the visualization of endogenous molecules.

## 4. Experimental Section

### 4.1. Drugs and Reagents

Paclitaxel (PTX, Indena S.p.A, Milan, Italy), ortataxel (OTX, Indena S.p.A, Milan, Italy), and imatinib (IMT, Norvartis, Basel, Switzerland) were dissolved in 50% ethanol, trabectedin (ET, PharmaMar Colmenar Viejo, Madrid, Spain) and lucitanib (LCT, Servier, Neully-sur-Seine, France) were dissolved in 50% methanol, and doxorubicin (DOXO, Nerviano Medical Science, Milan, Italy) was dissolved in H_2_O, all at a concentration of 100 pmol/µL.

For mouse treatments, PTX was dissolved in 50% Cremophor EL (Sigma Aldrich, Saint Louis, MO, USA) and 50% ethanol and further diluted in saline immediately before use. IMT and LCT were suspended in 0.5% Methocel.

### 4.2. Adopted Nanomaterials

Gold nanoparticles (AuNPs) of 20 nm diameter were synthesized as follows: an aqueous solution of HAuCl_4_ (1% *w*/*v*, 50 mL) was heated to a boil while stirring. Then trisodium citrate (0.75 mL, 1% *w*/*v*) was added quickly to the boiling mixture. The solution was refluxed for 15 min, and then allowed to cool to room temperature. Subsequently, the gold colloid was centrifuged at 13.2 krpm for 20 min and resuspended in phosphate buffered saline (PBS), pH 7.4. The average diameter of the AuNPs was determined by UV, TEM, SEM, and DLS.

Two different titanium nanoparticles, TiO_2_ P25 and TiO_2_ Hombikat UV100, were purchased from Evonik (Essen, Germany) and Sachtleben Chemie GmbH (Duisburg, Germany), respectively. Multiwalled Carbon Nanotubes (CNTs) were purchased from NANOCYL^®^ (NC7000™ series) and were produced by a Catalytic Chemical Vapor Deposition (CCVD) process. Halloysite nanoclay was purchased from Sigma Aldrich (Saint Louis, MO, USA). P25 or Hombikat TiO_2_, AuNPs, and HNTs or CNTs matrices suspensions were prepared respectively at 1 mg/mL, 0.4 mM, and 0.15 mg/mL in 50% ethanol, and were vortexed and sonicated before use.

### 4.3. Sample Characterization

UV-Visible measurements were recorded using a Hitachi UH 5300 Spectrophotometer (Hitachi High-Technologies Corporation Europe, Maidenhead, UK), equipped with 1.0 cm path length quartz cells and recorded in a range of 400-700 nm. The morphology and shape of the samples have been evaluated by means of a Zeiss Evo50 Scanning Electron Microscope (SEM, Zeiss, Germany) with an accelerating voltage of 20 kV and a magnification of 24000 × TEM analyses were performed on a Jeol Jem 3010 UHR instrument (JEOL, München, Germany).

Diffuse reflectance spectra (DRS) of the powders were measured on a UV-Vis scanning spectrophotometer (Perkin-Elmer, Waltham, MA, USA) equipped with a diffuse reflectance accessory. A ‘‘total white’’ Perkin Elmer reference material was used as a reference.

### 4.4. Cell Lines and Animal Models

Procedures involving animals and their care were conducted in conformity with the following laws, regulations, and policies governing the care and use of laboratory animals: Italian Governing Law (D.lgs 26/2014; Authorization n.19/2008-A issued 6 March 2008 by Ministry of Health); Mario Negri Institutional Regulations and Policies providing internal authorization for persons conducting animal experiments (Quality Management System Certificate—UNI EN ISO 9001:2008—Reg. N. 8576-A); the NIH Guide for the Care and Use of Laboratory Animals (2011 edition) and EU directives and guidelines (EEC Council Directive 2010/63/UE) and in line with Guidelines for the welfare and use of animals in cancer research [45]. The statement of Compliance (Assurance) with the Public Health Service (PHS) Policy on Human Care and Use of Laboratory Animals has been recently reviewed (9/9/2014) and will expire on 30 September 2019 (Animal Welfare Assurance #A5023-01).

Animal experiments have been reviewed and approved by the IRFMN Animal Care and Use Committee (IACUC) that includes members ad hoc for ethical issues. Animals were housed in the Institute’s Animal Care facilities which meet international standards. They were regularly checked by a certified veterinarian who is responsible for health monitoring, animal welfare supervision, experimental protocols, and procedures revision.

Six to seven-week-old female NCr-nu/nu mice (from the Harlan Lab) were inoculated subcutaneously with 10^7^ A2780 cells or fragments of the rare MPM487 human malignant pleural mesothelioma (obtained from the Mesothelioma Biobank-Pathology Dept, SS Antonio e Biagio General Hospital, Alessandria, Italy). Tumor growth was measured with a digital caliper two/three times a week and the tumor volume (mm^3^) was calculated as (length (mm) × width^2^ (mm^2^))/2.

When tumors reached approximately 500 mg, the animals were treated with different drugs or with the vehicle as a negative control. Mice bearing xenografts were treated with vehicle (CTRL) or a single dose of drug (60 mg/kg i.v. for PTX, 400 mg/kg for IMT p.o., 20 mg/kg for LCT p.o.). Animals were sacrificed 1 hour after treatment (4 h for PTX) under CO_2_ and all efforts were made to minimize suffering.

Tumors and organs were explanted, then immediately snap-frozen in liquid nitrogen and stored at −80 °C until analysis.

### 4.5. Mass Spectrometry

The different nanomaterials tested as matrices were firstly screened by spotting 0.5 µL of 100 pmol/µL drug standard solutions on the MALDI steel plate. After complete drying in air of the drug standard spots, 0.5 µL of matrix suspensions described above were spotted on top of the different drugs samples. Matrices were also tested on tissues to evaluate the influence of the biological matrix on the ion signal intensity, following the MSI protocol we recently published [22]. Briefly, frozen tissues were cut into 10 µm thick sections using a cryo-microtome (Leica Microsystems, Wetzler, Germany) at 20 °C and mounted on pre-cooled MALDI plates (Opti-TOF 384 Well insert) by standard thaw-mounting techniques. One section every 300 µm was cut from the central part of the control and the treated tumors. The plates were then dried overnight in a vacuum drier at room temperature. Different drug standards were spotted on control tumor sections, then each plate was sprayed with different matrix suspensions using a BD 180 precision double-action trigger airbrush (Fenga, Mexico) with a 0.20 mm nozzle diameter, using nitrogen at 0.2 atm. Care was taken to avoid over-spraying the matrix suspension on a single point so as to avoid the formation of droplets that would wet the surface with possible damage of the tissue structure, analyte diffusion, or partial detachment of the slice from the plate.

A MALDI 4800 TOF-TOF (AB SCIEX Old Connecticut Path, Framingham, MA, USA) was used, equipped with a 355 nm Nd:YAG laser with a 200 Hz repetition rate, controlled by the 4000 Series Explorer TM software (AB SCIEX Old Connecticut Path, Framingham, MA, USA). MS spectra were acquired with 20 laser shots with an intensity of 6000 arbitrary units, with a bin size of 1.0 ns, acquiring spectra in reflectron, both in negative and positive-ion mode. Images of tissue sections were acquired using the 4800 Imaging Tool Software (www.maldi-msi.org, M. Stoeckli, Novartis Pharma, Basel, Switzerland) with an imaging raster of 100 µm. Tissue View software 1.1 (AB SCIEX Old Connecticut Path, Framingham, MA, USA) was used to process and display the ion distributions inside the tumor sections. The ions plotted for each drug are reported in Appendix A (Figure A1, Figure A2, Figure A3, Figure A4, Figure A5 and Figure A6).

## 5. Conclusions

Testing different types of materials has made it possible to ionize molecules with different molecular weights and chemical properties, overcoming the problem of high background noise in the low mass range that is typical in classic MALDI experiments. Moreover, with these nanomaterials we could visualize the mitotic inhibitor PTX and the tyrosine kinase inhibitor IMT inside tumors harvested from treated mice.

## Figures and Tables

**Table 1:** Physico-chemical characteristics of all tested nanomaterials.

	*d*_TEM_/nm	*S*_BET_/(m^2^·g^−1^)	*V*_pores_/(mL·g^−1^)
Au nanoparticles	20 ± 2	n.d.	n.d.
TiO_2_ HOMBIKAT nanoparticles	7 ± 2	350	0.26
TiO_2_ P25 nanoparticles	24 ± 6	52	0.27
Halloysite	Average diameter: 30–70 Inner lumen diameter: 15 Average Length: 500–2000	65 *	1.25 *
Carbon nanotubes *	Average diameter: 9.5 Average Length: 1500	250–300	n.a.

Notes: n.d. not determinable; n.a. not available; * from technical data sheets.
